# Drug survival of biologic treatments for psoriasis and psoriatic arthritis in Denmark, 2018–23: a nationwide register–based cohort study

**DOI:** 10.1093/skinhd/vzag054

**Published:** 2026-05-04

**Authors:** Sejun Kim, Andreas Jensen, Alexander Egeberg, Lone Graff Stensballe

**Affiliations:** Department of Paediatrics and Adolescents Medicine, Rigshospitalet, Copenhagen, Denmark; Mary Elizabeth’s Hospital’s Data Powerhouse, Rigshospitalet, Copenhagen, Denmark; Department of Clinical Medicine, Faculty of Health and Medical Sciences, University of Copenhagen, Copenhagen, Denmark; Department of Paediatrics and Adolescents Medicine, Rigshospitalet, Copenhagen, Denmark; Mary Elizabeth’s Hospital’s Data Powerhouse, Rigshospitalet, Copenhagen, Denmark; Department of Clinical Medicine, Faculty of Health and Medical Sciences, University of Copenhagen, Copenhagen, Denmark; Department of Dermatology, Bispebjerg Hospital, Copenhagen, Denmark; Department of Paediatrics and Adolescents Medicine, Rigshospitalet, Copenhagen, Denmark; Mary Elizabeth’s Hospital’s Data Powerhouse, Rigshospitalet, Copenhagen, Denmark; Department of Clinical Medicine, Faculty of Health and Medical Sciences, University of Copenhagen, Copenhagen, Denmark

## Abstract

**Background:**

Psoriasis is a chronic inflammatory skin condition. It is being increasingly treated with biologic agents targeting specific immune pathways. These treatments target specific immune system pathways involved in the disease. Drug survival (how long patients remain on a given treatment) is a key measure of long-term effectiveness and safety.

**Objectives:**

To analyse the drug survival rates of approved biologics for psoriasis using Danish nationwide register-based data from 2018 to 2023.

**Methods:**

This study used a nationwide register-based cohort of patients diagnosed with psoriasis in Denmark who were treated with biologics between 2018 and 2023. Patients were stratified by the presence or absence of psoriatic arthritis (PsA). Drug survival was assessed using Kaplan–Meier curves, and the rate of discontinuation was analysed using Cox proportional hazards regression models.

**Results:**

Of the biologics studied, infliximab had the highest 1-year drug survival (49%), followed by ustekinumab (42%). Bimekizumab, the most recently introduced biologic in Denmark, had a 19% drug survival rate after 349 days. Ustekinumab had longer persistence in patients who only had psoriasis, but the drug survival rate was reduced in patients with PsA. In contrast, infliximab maintained robust performance across both patient groups, with a significantly lower risk of discontinuation compared with ustekinumab (hazard ratio 0.59, 95% confidence intervals 0.53–0.67), particularly in patients with PsA.

**Conclusions:**

Drug survival differed across biologic treatments and according to PsA status. Infliximab had higher overall drug survival, particularly in patients with PsA. Ustekinumab had greater treatment persistence in patients without PsA but lower drug survival in those with PsA.

## Teaser

Biologic treatments have advanced the management of psoriasis, but drug survival remains a key indicator of long-term effectiveness and safety. Using Danish registry data from 2018 to 2023, we assessed biologic survival in patients with psoriasis, stratified by psoriatic arthritis, with Kaplan–Meier and Cox regression analyses. Infliximab had the highest 1-year survival (49%) and outperformed ustekinumab, particularly in those with psoriatic arthritis (hazard ratio 0.59, 95% confidence intervals 0.53–0.67). Ustekinumab had better persistence in patients with psoriasis only. These findings show higher treatment persistence for infliximab compared with other biologics.

What is already known about this topic?

- Psoriasis is a common chronic inflammatory skin disease driven by immune system dysfunction.
- In recent decades, biologic treatments targeting cytokine signalling pathways have significantly improved patient outcomes.
- Evaluating drug survival is essential for assessing the long-term effectiveness and safety of biologic treatments.
- In Denmark, no comprehensive studies have compared drug survival of the biologics available to treat psoriasis since the introduction of interleukin-23 inhibitors.
- This study used nationwide Danish register data (2018–23) to assess drug survival of biologics across treatment episodes for psoriasis in a real-world setting.

What does this study add?

- Infliximab has the highest 1-year drug survival (42%), followed by ustekinumab (33%).
- Bimekizumab, the most recently introduced biologic in Denmark, has a drug survival rate of 19% at 1 year.
- Infliximab has greater persistence across patients with psoriasis and psoriatic arthritis (PsA), with a lower risk of discontinuation than ustekinumab.
- Drug survival of ustekinumab varies by PsA status.
- Patients with PsA had 10% lower drug survival, revealing reduced effectiveness in this subgroup.

Psoriasis is a chronic inflammatory skin disorder driven by immune system dysfunction. It affects around 5% of the Danish population.^1–3^ Over the past 20 years, the use of biologics, including tumour necrosis factor (TNF)-α and interleukin (IL) inhibitors, has become central to the management of psoriasis.^4^ The identification of the T helper 17 pathway as a critical driver of psoriasis pathogenesis has led to the development and introduction of IL-17- and IL-23-targeted antibodies in Denmark, which have higher skin clearance rates than earlier biologics.^5–9^

Drug survival, defined as the time from initiation to discontinuation (e.g. treatment switching to another biologic), serves as an important real-world indicator of a biologic’s long-term effectiveness, safety and tolerability. This metric is essential for optimizing treatment strategies.^9–12^ However, few comprehensive studies have evaluated and compared the drug survival of all the biologics available in Denmark to treat psoriasis since the introduction of the first IL-23 inhibitor in 2018.^2,12^ This study is among the first nationwide analyses in Denmark to include multiple IL-23 inhibitors with follow-up through to 2023. Using Danish register-based data from 2018 to 2023, this study addresses this gap by analysing the drug survival rates of approved biologics.

## Materials and methods

### Data sources

This study used data from a number of Danish national databases, including the National Patient Register (NPR), the National Prescription Register and the population register.^13–15^ A key feature of the Danish data infrastructure is the Central Person Register (CPR) number, which is assigned to all Danish residents at birth or upon immigration.^16^ This unique identifier enables the integration of data across different national registers, facilitating comprehensive longitudinal studies. The CPR number remains consistent throughout an individual’s life, ensuring reliable long-term follow-up across national registers.

The population registers provide information on residency, occupation, emigration and immigration. The NPR contains detailed records of all inpatient and outpatient visits to specialist care, including primary and secondary diagnoses at discharge and procedure codes. Psoriasis and psoriatic arthritis (PsA) diagnoses were recorded using the International Classification of Diseases, 10th Revision (ICD-10).^17^ The National Prescription Registry includes data on treatment formulations and dispensing dates, with medications classified using the World Health Organization Anatomical Therapeutic Chemical (ATC) classification system.^18^

### Population

The present study analysed data from the Danish national health, administrative and socioeconomic registers, covering a population of around 6 million individuals. The focus was on individuals with hospital-diagnosed psoriasis from 1994 to 2023, identified using ICD-10 codes L40, M07.0, M07.1, M07.2, M07.3 and M09.0, including all relevant subcodes.^19,20^ The development of PsA was tracked using ICD-10 codes L40.5, M07.0, M07.1, M07.2, M07.3 and M09.0, including all subcodes.^20,21^

### Exposure

The study included biologic treatments administered to patients in Denmark between 2018 and 2023. The treatments were categorized based on the targeted molecules, including IL-12/IL-23 inhibitors (ustekinumab), TNF-α inhibitors (adalimumab, certolizumab, etanercept and infliximab), IL-17 inhibitors (bimekizumab, brodalumab, ixekizumab and secukinumab) and IL-23p19 (IL-23) inhibitors (guselkumab and tildrakizumab). Risankizumab (an IL-23 inhibitor) was included in the analyses, but the results were not reported, in accordance with the terms of the research agreement with its manufacturer (AbbVie). Each treatment was identified by its ATC code or a specific procedure code for in-hospital administration.^15^ A comprehensive list of these codes is provided in Table S1 (see Supporting Information).

### Study design

This nationwide population-based cohort study included a 6-year follow-up period, spanning from 2018 to 2023, to minimize bias introduced by evolving therapeutic policies. Ustekinumab was approved as a first-line treatment for psoriasis in 2012, IL-17 inhibitors were introduced in 2015 and IL-23 inhibitors after 2018, substantially altering treatment pathways. Focusing on the period after IL-23 inhibitors were introduced for psoriasis treatment in Denmark enabled comparisons across all major treatment classes. Treatment episodes with specific biologics were considered discontinued if patients switched to another biologic or if a treatment gap exceeded the defined interval, consisting of the standard dosing duration plus a 90-day grace period. The intervals were defined as follows based on the defined daily doses (DDDs): 1 month (4 weeks) for adalimumab, certolizumab and etanercept; 2 months (8 weeks) for brodalumab, guselkumab, infliximab, ixekizumab, secukinumab and tildrakizumab; and 3 months (12 weeks) for bimekizumab and ustekinumab. Treatment episodes were censored at the time of emigration, death or upon reaching 31 December 2023. Only treatment episodes initiated within the study period were included in the analysis.

Treatment episodes among bionaïve users (i.e. individuals with no prior exposure to any biologic treatment for psoriasis before their first administered treatment episode within the study period) were considered in a supplementary analysis (Table S2, Figure S1; see Supporting Information).

Additional supplementary analyses separated results by treatment series into first, second, third or more, starting from the introduction of the first TNF inhibitors in Denmark in 2004. The drug survival results stratified by treatment series are presented in Table S3 and Figure S2 (see Supporting Information).

### Statistical modelling and data interpretation

Drug survival was assessed with Kaplan–Meier curves, while Cox proportional hazards regression models were applied to evaluate the relative drug discontinuation rates, defined as the hazard of treatment termination or switching to an alternative biologic. Hazard ratios (HRs) were estimated to compare the discontinuation rates between ustekinumab (reference treatment) and other treatments, both specific treatments and treatment groups. The Cox models were adjusted for PsA status.^22^

### Subgroup analyses

Patients were divided into two subgroups: those with a confirmed PsA diagnosis (hereafter ‘with PsA’) and those without PsA at the first treatment episode administration date (‘without PsA’). Individuals who were also diagnosed with PsA after entering the study were included in the ‘with PsA’ subgroup from the time of their PsA diagnosis and were analysed independently in both subgroups. All analyses were conducted using the same methodology as applied in the overall analyses. The differences in drug survival rates for each treatment between subgroups were statistically assessed using log-rank test (Table S4; see Supporting Information). The detailed baseline characteristics are presented in Table S5 (see Supporting Information).

### Statistical analysis

Statistical analyses were conducted in R (version 4.4.1; R Foundation for Statistical Computing, Vienna, Austria) on a Windows 10 platform. Data manipulation was carried out using the ‘tidyverse’ (version 2.0.0) and ‘haven’ (version 2.5.4) packages,^23,24^ while survival modelling was done using the ‘survival’ package (version 3.7.0).^25^ Data visualization was performed with the ‘ggplot2’ package (version 3.5.1).^26^

## Results

### Baseline characteristics

Over the 6-year follow-up period (2018–23), distinct patterns in drug survival were observed across treatments. Adalimumab was the most frequently used treatment, with 6694 unique patients contributing to 15 785 treatment episodes (Table 1). Etanercept followed, with 1857 patients and 4464 episodes. Secukinumab and ustekinumab also exhibited substantial use, each involving >1300 patients and exceeding 2200 treatment episodes.

**Table 1:** Number of unique patients and treatment episodes for each treatment, 2018–23

Treatment group	Treatment	Unique patients (*n*)	Treatment episodes (*n*)
Ustekinumab	Ustekinumab	1308	2213
TNF inhibitors	Adalimumab	6694	15 785
	Certolizumab	455	897
	Etanercept	1857	4464
	Infliximab	1083	1221
IL-17 inhibitors	Bimekizumab	140	145
	Brodalumab	262	521
	Ixekizumab	954	1744
	Secukinumab	1312	3102
IL-23 inhibitors	Guselkumab	269	555
	Tildrakizumab	18	52

IL, interleukin; TNF, tumour necrosis factor.

### Drug survival analyses by each treatment and treatment group

**Figure 1:**
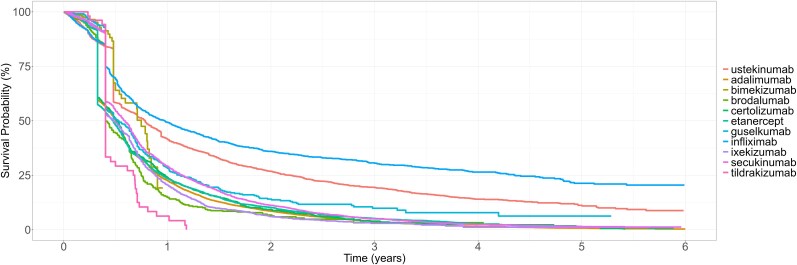
Kaplan–Meier curves of drug survival for each treatment with 6 years of follow-up.

**Figure 2:**
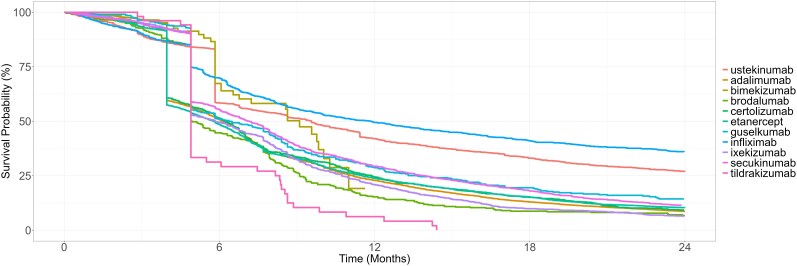
Kaplan–Meier curves drug survival for each treatment with 2 years of follow-up.

Throughout the follow-up period, infliximab maintained the highest drug survival rate, with 49% survival at 1 year (Figures 1, 2; Table 2). Ustekinumab followed, with a 42% 1-year survival rate. Bimekizumab, the most recently introduced treatment in Denmark, exhibited a 19% drug survival rate at the maximum ­follow-up duration of 349 days. Infliximab and ustekinumab sustained drug survival rates of >25% after 2 years, whereas all other biologics had survival rates of ≤14% after 2 years.

**Table 2:** Drug survival by treatment, 2018–23

Treatment group	Treatment	Drug survival rate (%) by time (years)				
		1	2	3	4	5
Ustekinumab	Ustekinumab	42	27	19	14	11
TNF inhibitors	Adalimumab	23	9	4	2	1
	Certolizumab	24	9	4	3	1
	Etanercept	23	10	5	3	1
	Infliximab	49	36	31	26	21
IL-17 inhibitors	Bimekizumab^a^	19				
	Brodalumab	15	7	3	3	
	Ixekizumab	21	6	3	1	1
	Secukinumab	29	11	5	2	1
IL-23 inhibitors	Guselkumab	28	14	10	8	6
	Tildrakizumab	6				

IL, interleukin; TNF, tumour necrosis factor. ^a^Bimekizumab was first used in Denmark in 2022 and had <1 year of follow-up. The maximum follow-up period of bimekizumab was 349 days.

**Figure 3:**
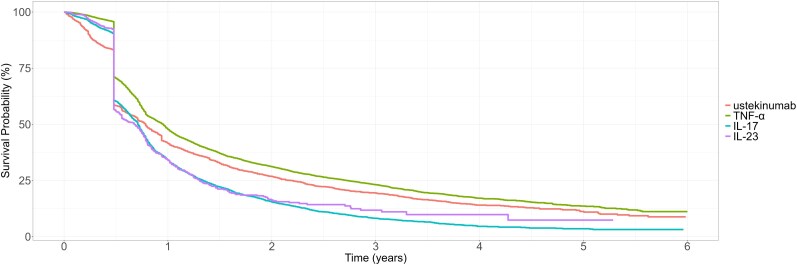
Kaplan–Meier curves of drug survival for each treatment group with 6 years of follow-up. IL, interleukin; TNF, tumour necrosis factor.

At the aggregated treatment-group level, TNF inhibitors and ustekinumab exhibited higher 1-year survival (42% and 48%, respectively), followed by IL-17 and IL-23 inhibitors (both 34%) (Figure 3, Table 3). TNF inhibitors had the highest drug survival rates across the entire study period.

**Table 3:** Drug survival by treatment group, 2018–23

Treatment group	Drug survival rate (%) by time (years)				
	1	2	3	4	5
Ustekinumab	42	27	19	14	11
TNF inhibitors	48	31	23	17	14
IL-17 inhibitors	34	15	8	5	4
IL-23 inhibitors	34	16	12	10	7

IL, interleukin; TNF, tumour necrosis factor.

The marked decrease in drug survival between 3 and 6 months was explained by the treatment episode construction: if no additional record of the drug was registered within the DDD period plus a 90-day grace period (118 days to 174 days), the treatment was considered discontinued at that point.

**Figure 4:**
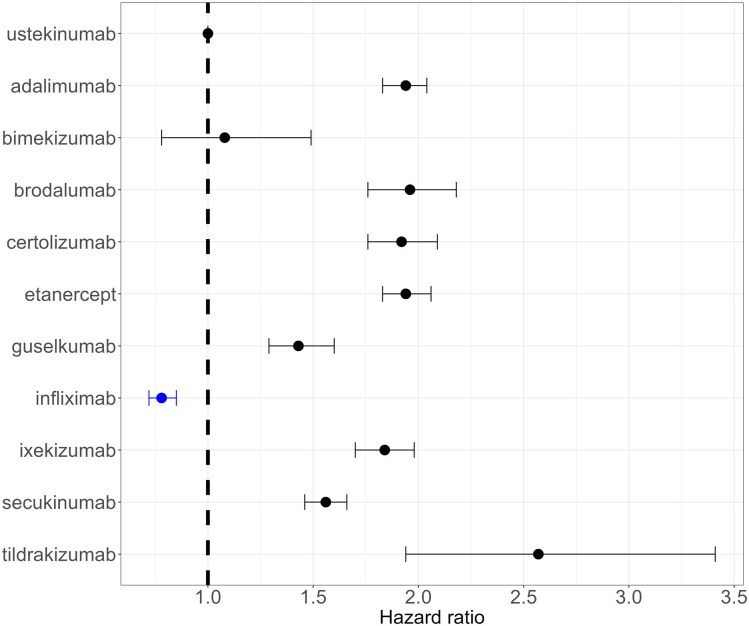
Hazard ratios (HRs) of drug discontinuation by comparator. The Cox regression model was fitted by each treatment adjusted for psoriatic arthritis status. Each dot represents the estimated HR comparing ustekinumab with each comparator treatment. The horizontal solid lines represent the 95% confidence interval. The black vertical dashed line indicates the point of identical discontinuation hazards between ustekinumab and each comparator. Black favours ustekinumab. Blue favours the comparator treatment.

**Figure 5:**
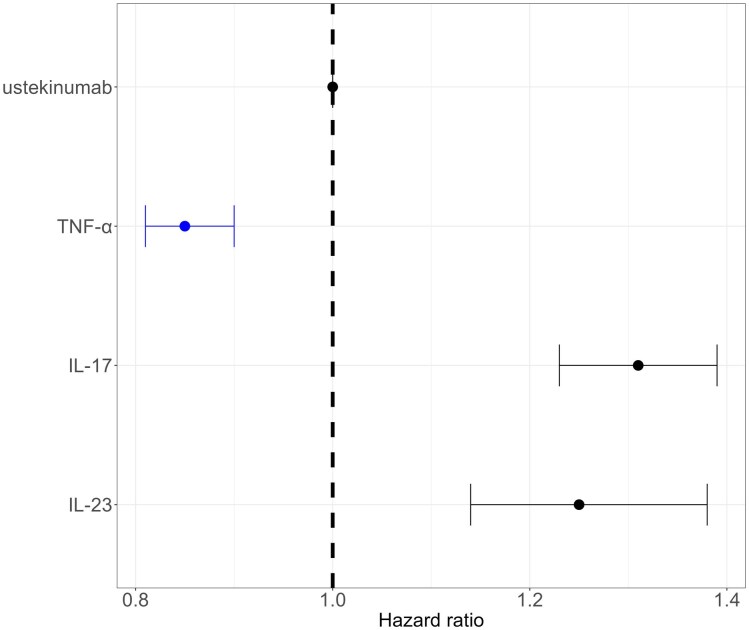
Hazard ratios (HRs) of treatment discontinuation by treatment group. The Cox regression model was fitted by each treatment group with psoriatic arthritis status. The dots represent the point estimate of HRs between ustekinumab and each comparator. The black horizontal lines represent the 95% confidence intervals. The black vertical dashed lines indicate the point of identical hazards between ustekinumab and each comparator. Black favours ustekinumab. Blue favours the comparator. IL, interleukin; TNF, tumour necrosis factor.

Cox proportional hazards models indicated that patients with psoriasis treated with infliximab had the highest drug survival rate compared with other treatments (Figure 4, Table 4), with a PsA ­status-adjusted HR of 0.78 [95% confidence interval (CI) 0.72–0.85] relative to ustekinumab. Other treatments were associated with a significantly higher rate of discontinuation: tildrakizumab (HR 2.57, 95% CI 1.94–3.41), brodalumab (HR 1.96, 95% CI 1.76–2.18) and adalimumab (HR 1.94, 95% CI 1.83–2.04). Notably, IL-17 inhibitors as a whole exhibited a higher discontinuation rate compared with ustekinumab (HR 1.31, 95% CI 1.23–1.39), while TNF inhibitors had the lowest discontinuation rate (HR 0.85, 95% CI 0.81–0.90) relative to ustekinumab (Figure 5, Table 4). No significant differences were found in the analyses restricted to the bionaïve population or when stratified by treatment series [Figures S1, S2; Tables S2, S3 (see Supporting Information)].

**Table 4:** Hazard ratios for discontinuation: ustekinumab vs. each comparator, 2018–23

Treatment group	HR (95% CI) for treatment group	Treatment	HR (95% CI) for each treatment
TNF inhibitors	0.85 (0.81–0.90)	Adalimumab	1.94 (1.83–2.04)
		Certolizumab	1.92 (1.76–2.09)
		Etanercept	1.94 (1.83–2.06)
		Infliximab	0.78 (0.72–0.85)
IL-17 inhibitors	1.31 (1.23–1.39)	Bimekizumab	1.08 (0.78–1.49)
		Brodalumab	1.96 (1.76–2.18)
		Ixekizumab	1.84 (1.70–1.98)
		Secukinumab	1.56 (1.46–1.66)
IL-23 inhibitors	1.25 (1.14–1.38)	Guselkumab	1.43 (1.29–1.60)
		Tildrakizumab	2.57 (1.94–3.41)

CI, 95% confidence interval; HR, hazard ratio; IL, interleukin; TNF, tumour necrosis factor.

### Subgroup drug survival analysis

**Figure 6:**
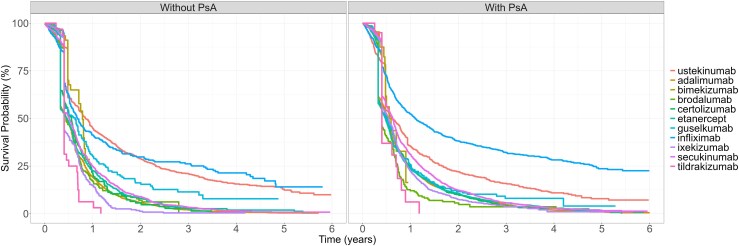
Kaplan–Meier curves of drug survival by treatment and psoriatic arthritis (PsA) status over 6 years of follow-up. ‘Without PsA’ refers to patients diagnosed with psoriasis but not diagnosed with PsA. ‘With PsA’ refers to patients diagnosed with psoriasis and diagnosed with PsA.

**Figure 7:**
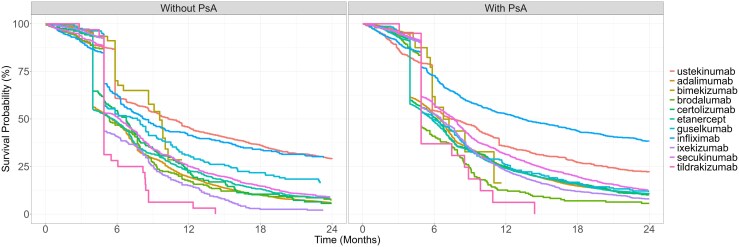
Kaplan–Meier curves of drug survival by treatment and psoriatic arthritis (PsA) status over 2 years of follow-up. ‘Without PsA’ refers to patients diagnosed with psoriasis but not diagnosed with PsA. ‘With PsA’ refers to patients diagnosed with psoriasis and diagnosed with PsA.

The survival rate of ustekinumab varied between subgroups. After 1 year, patients without PsA had a survival rate of 45% compared with 35% for those with PsA (Figures 6; 7; Table 5). Throughout the follow-up period, the ‘without PsA’ group consistently had higher ustekinumab survival than the ‘with PsA’ group (Table S3; see Supporting Information).

**Table 5:** Drug survival for each treatment by psoriatic arthritis status, 2018–23

Treatment group	Treatment	Subgroup^a^	Drug survival rate (%) by time (years)				
			1	2	3	4	5
Ustekinumab	Ustekinumab	Without PsA	45	29	21	16	13
		With PsA	35	22	16	11	8
TNF inhibitors	Adalimumab	Without PsA	19	6	2	1	0
		With PsA	25	10	5	2	1
	Certolizumab	Without PsA	21	6	3		
		With PsA	24	10	4	3	1
	Etanercept	Without PsA	22	8	3	2	2
		With PsA	24	11	6	3	1
	Infliximab	Without PsA	41	30	26	21	14
		With PsA	52	38	32	28	24
IL-17 inhibitors	Bimekizumab^b^	Without PsA	29				
		With PsA	16				
	Brodalumab	Without PsA	17	7			
		With PsA	12	6	4	4	
	Ixekizumab	Without PsA	15	1	0	0	
		With PsA	22	7	4	1	1
	Secukinumab	Without PsA	25	9	3	1	1
		With PsA	31	12	6	3	2
IL-23 inhibitors	Guselkumab	Without PsA	30	16	11	8	
		With PsA	26	12	8	8	4
	Tildrakizumab	Without PsA	6				
		With PsA	6				

IL, interleukin; PsA, psoriatic arthritis; TNF, tumour necrosis factor. ^a^‘Without PsA’ refers to patients diagnosed with psoriasis but not diagnosed with PsA; ‘with PsA’ refers to patients diagnosed with psoriasis and diagnosed with PsA. ^b^Bimekizumab was first used in Denmark in 2022 and had <1 year of follow-up. The maximum follow-up period of bimekizumab was 343 and 349 days for the ‘without PsA’ and ‘with PsA’ groups, respectively.

**Figure 8:**
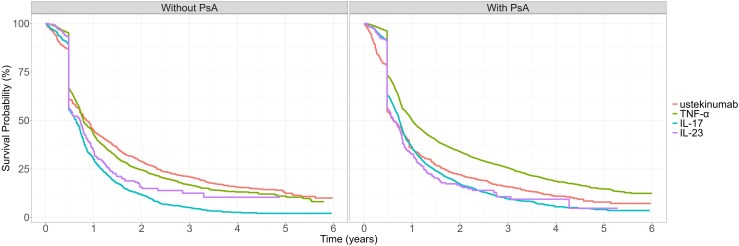
Kaplan–Meier curves of the 6-year drug survival rate for each treatment group by psoriatic arthritis (PsA) status. ‘Without PsA’ refers to patients diagnosed with psoriasis but not diagnosed with PsA. ‘With PsA’ refers to patients diagnosed with psoriasis and diagnosed with PsA. IL, interleukin; TNF, tumour necrosis factor.

Infliximab also had high drug survival regardless of PsA status, maintaining the highest rates over 6 years, especially in the population of patients with psoriasis and PsA, with 1-year survival rates of 41% in the ‘without PsA’ group and 52% in the ‘with PsA’ group (Table S3; see Supporting Information). At the treatment-group level, ustekinumab consistently had the highest drug survival in patients without PsA. Ustekinumab had the highest 1-year drug survival (45%), followed by TNF inhibitors (43%), IL-23 inhibitors (34%) and IL-17 inhibitors (30%) (Figure 8, Table 6). Of those patients with psoriasis and PsA, TNF inhibitors had superior survival rates to other treatment groups (50% after 1 year), followed by IL-17 inhibitors (36%), ustekinumab (35%) and IL-23 inhibitors (33%).

**Table 6:** Drug survival rate for each treatment group by psoriatic arthritis status, 2018–23

Treatment group	Subgroup^a^	Drug survival rate (%) by time (years)				
		1	2	3	4	5
Ustekinumab	Without PsA	45	29	21	16	13
	With PsA	35	22	16	11	8
TNF inhibitors	Without PsA	43	24	17	13	11
	With PsA	50	34	26	19	15
IL-17 inhibitors	Without PsA	30	12	5	3	2
	With PsA	36	17	10	5	4
IL-23 inhibitors	Without PsA	34	16	12	10	
	With PsA	33	16	11	9	5

IL, interleukin; PsA, psoriatic arthritis; TNF, tumour necrosis factor. ^a^‘Without PsA’ refers to patients diagnosed with psoriasis but not diagnosed with PsA; ‘with PsA’ refers to patients diagnosed with psoriasis and diagnosed with PsA.

**Figure 9:**
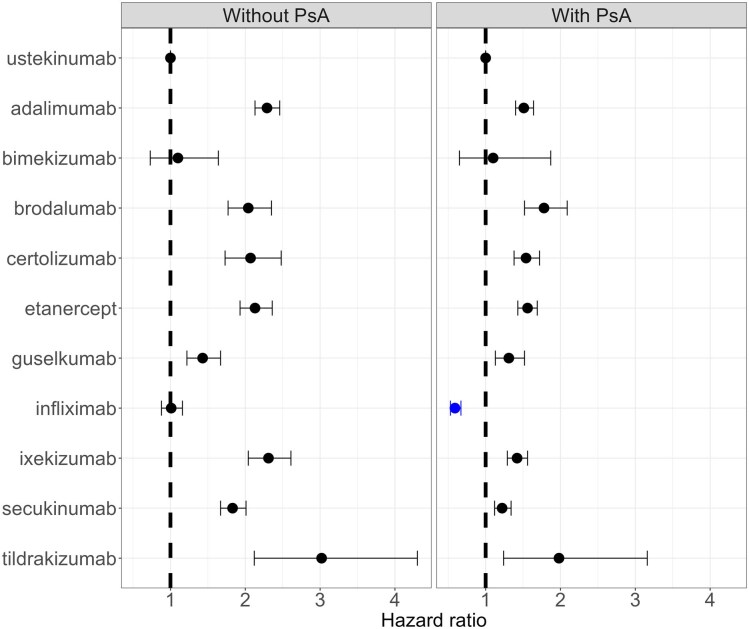
Hazard ratios (HRs) by comparator and psoriatic arthritis (PsA) status. ‘Without PsA’ refers to patients diagnosed with psoriasis but not diagnosed with PsA. ‘With PsA’ refers to patients diagnosed with psoriasis and diagnosed with PsA. Each dot represents the estimated HR comparing ustekinumab with another treatment. The black horizontal lines represent the 95% confidence intervals. The black vertical dashed line indicates the point of identical hazards between ustekinumab and each comparator. Black favours ustekinumab or equivalent with ustekinumab. Blue favours the comparator treatment.

Based on the Cox regression model, infliximab showed similar or lower discontinuation rates in both subgroups compared with ustekinumab, with HRs of 1.01 (95% CI 0.88–1.16) and 0.59 (95% CI 0.53–0.67) for the ‘without PsA’ and ‘with PsA’ groups, respectively (Figure 9, Table 7).

**Table 7:** Hazard ratio (HR) between discontinuation of ustekinumab and each comparator by psoriatic arthritis status, 2018–23

Treatment group	HR (95% CI) for treatment group		Treatment	HR (95% CI) for each treatment	
	Without PsA^a^	With PsA		Without PsA	With PsA
TNF inhibitors	1.03 (0.96–1.11)	0.64 (0.59–0.70)	Adalimumab	2.29 (2.13–2.46)	1.51 (1.40–1.64)
			Certolizumab	2.07 (1.73–2.48)	1.54 (1.38–1.72)
			Etanercept	2.13 (1.93–2.36)	1.56 (1.43–1.69)
			Infliximab	1.01 (0.88–1.16)	0.59 (0.53–0.67)
IL-17 inhibitors	1.54 (1.41–1.68)	1.00 (0.92–1.09)	Bimekizumab	1.10 (0.73–1.64)	1.10 (0.65–1.87)
			Brodalumab	2.04 (1.77–2.35)	1.78 (1.52–2.09)
			Ixekizumab	2.31 (2.04–2.61)	1.42 (1.29–1.56)
			Secukinumab	1.83 (1.67–2.01)	1.22 (1.12–1.34)
IL-23 inhibitors	1.31 (1.14–1.50)	1.09 (0.95–1.26)	Guselkumab	1.43 (1.22–1.67)	1.31 (1.13– 1.52)
			Tildrakizumab	3.02 (2.12–4.30)	1.98 (1.24–3.16)

CI, confidence interval; IL, interleukin; PsA, psoriatic arthritis; TNF, tumour necrosis factor. ^a^‘Without PsA’ refers to patients diagnosed with psoriasis but not diagnosed with PsA; ‘with PsA’ refers to patients diagnosed with psoriasis and diagnosed with PsA.

**Figure 10:**
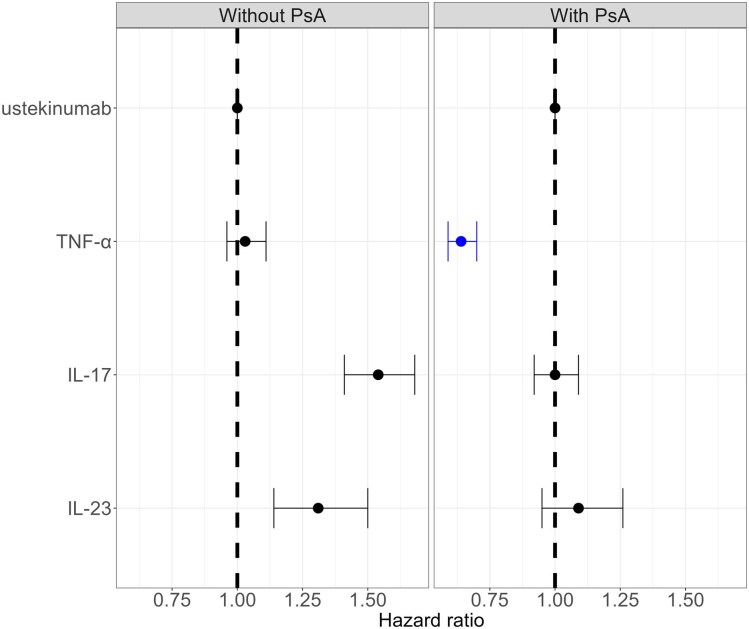
Hazard ratios (HRs) by treatment group and psoriatic arthritis (PsA) status. ‘Without PsA’ refers to patients diagnosed with psoriasis but not diagnosed with PsA. ‘With PsA’ refers to patients diagnosed with psoriasis and diagnosed with PsA. The dots represent the point estimate of HRs between ustekinumab and each comparator. The black horizontal lines represent the 95% confidence intervals. The black vertical dashed lines indicate the point of identical hazards between ustekinumab and each comparator. Black favours ustekinumab. Blue favours the comparator.

At the treatment-group level, ustekinumab had the lowest discontinuation rate among patients with psoriasis without PsA, followed by TNF inhibitors (HR 1.03, 95% CI 0.96–1.11). Meanwhile, in the group of patients with psoriasis with PsA, TNF inhibitors had a lower discontinuation risk than ustekinumab (HR 0.64, 95% CI 0.59–0.70) (Figure 10, Table 7).

## Discussion

In this nationwide cohort study with six full calendar years of ­follow-up (2018–23), infliximab had the highest drug survival among Danish patients with hospital-diagnosed psoriasis. This is probably due to its superior effectiveness, safety and affordability following the introduction of biosimilars.^12,27^ Ustekinumab ranked second in terms of drug survival; however, its persistence varied according to whether the patient also had PsA. This variation may reflect reduced efficacy or safety of ustekinumab in patients with both psoriasis and PsA compared with those with psoriasis only.

In this study, adalimumab, etanercept, infliximab, secukinumab and ustekinumab were identified as the most commonly used biologic treatments for psoriasis in Denmark over the past 6 years. Notably, the use of etanercept and infliximab, both TNF inhibitors, increased significantly after 2018.^28^ This trend coincided with the introduction of biosimilars from 2015, which reduced treatment costs and improved accessibility.^29–31^

During the 6-year follow-up period, infliximab and ustekinumab had the highest drug survival rates among all biologic treatments for psoriasis. Ustekinumab has been the recommended first-line treatment for psoriasis in Denmark since 2012, largely due to its established safety profile, efficacy and cost-effectiveness.^32,33^ These factors probably contributed to its relatively low discontinuation rates. However, infliximab achieved the highest overall drug survival rate across the study period, suggesting superior safety, efficacy or both compared with ustekinumab. The increased survival of infliximab may also be attributed to the introduction of its biosimilars, which significantly reduced its cost, encouraging greater and sustained use by healthcare providers.^27,28^ Specifically, infliximab treatment is administered intravenously by medical professionals, requiring patients to visit the hospital regularly. In contrast, other biologic treatments taken at home or with flexible dosing schedules may be under-reported, as patients can extend treatment intervals or discontinue use without clinical documentation. This can lead to gaps in recording and imprecise estimation of actual drug survival. Additionally, discrepancies may arise from manual data entry into healthcare registries, leading to misclassification. However, register data generally maintain high diagnostic accuracy.^19,28^ Overall, TNF inhibitors, including infliximab, exhibited slightly higher drug survival. Conversely, biologics targeting the IL-17 and IL-23 pathways had lower survival rates than reported in previous studies.^9,34,35^

The findings of the present study diverge from drug survival studies from France and the UK, and an earlier report from Denmark, which identified higher survival of ustekinumab and newer IL-23 inhibitors.^28,34,36–38^ Overall drug survival in this cohort was lower than previously reported in other studies based on datasets such as Dermbio.^28^ Unlike administrative registers, such as the Danish NPR, Dermbio directly records treatment discontinuation dates, eliminating the need to estimate follow-up durations for treatment episodes.^28^ According to previous research, drug survival estimates derived from Dermbio are generally higher than those from the Danish NPR.^28^ This discrepancy is likely due to structural differences between the data sources and the potential for misclassification caused by incomplete manual reporting of discontinuation events, which tends to result in an overestimation of drug survival rates.^28^ The results of the present study are consistent with previous analyses based on the Danish NPR,^28^ supporting the view that these findings accurately reflect real-world drug use patterns in Denmark.

Subgroup analyses revealed variation in the drug survival of ustekinumab depending on the presence of PsA (Table S3; see Supporting Information). Patients with psoriasis alone exhibited approximately 10% higher drug survival than those with concurrent psoriasis and PsA. This difference is consistent with existing evidence indicating the reduced efficacy of ustekinumab in managing PsA compared with psoriasis.^32,39,40^

In contrast, TNF inhibitors appeared to bridge this efficacy gap in patients with PsA. In this subgroup, infliximab had the highest drug survival, regardless of PsA status, further supporting its role as a robust treatment option for both psoriasis and PsA. Other TNF inhibitor treatments also had longer persistence in patients with PsA than in those without PsA, underscoring their importance in addressing the specific needs of this subgroup.

A key strength of this study is its comprehensive analysis of drug discontinuation across all biologic target groups for psoriasis treatment in Denmark, including TNF inhibitors, an IL-12/IL-23 inhibitor (ustekinumab), IL-17 inhibitors and IL-23 inhibitors. This analysis is based on the longest real-world data period available, providing robust insights into treatment persistence. Additionally, the study captures real-world treatment patterns and further evaluates treatment use profiles in subgroups of patients with psoriasis stratified by PsA status, enhancing the understanding of treatment in different patient populations.

A limitation of this study is the data collection process, which relied on hospital-based psoriasis diagnoses and the administration of biologic treatments in Denmark. As hospital care typically involves patients with more severe symptoms, this may have led to an overestimation of the proportion of patients with psoriasis with PsA. In addition, drug survival definitions based on administration frequency and DDD may favour treatments with longer dosing intervals, potentially inflating survival estimates compared with more frequently administered treatments. Notably, since 2021, Dermbio has been unavailable to external investigators; consequently, Dermbio data could not be used in this study.

Additionally, although risankizumab (IL-23 inhibitor) was included in the analyses and contributed to treatment discontinuation outcomes, including discontinuations due to treatment switching from other treatments, risankizumab-specific results are not presented due to a research agreement with the manufacturer. Consequently, the absence of risankizumab-specific reporting may have led to an underestimation of the true total number of patients.

This study identified infliximab and ustekinumab as the biologics with the highest drug survival among patients in Denmark with hospital-diagnosed psoriasis over a 6-year period. Ustekinumab had superior longevity in patients without PsA but reduced persistence in those with PsA, reflecting its lower efficacy in this subgroup.^32,39,40^ The drug survival of TNF inhibitors, particularly infliximab, was robust across psoriasis and PsA groups, likely due to their strong efficacy and cost-effectiveness following the introduction of biosimilars.

## Supplementary Material

vzag054_Supplementary_Data

## Data Availability

Access to the data is restricted to authorized researchers through Statistics Denmark. Therefore, the present data collection is not available to others, but similar data collections can be established by other researchers with Statistics Denmark authorization.
