# Supplementary material for: Drug survival of biologic treatments for psoriasis and psoriatic arthritis in Denmark, 2018–23: a nationwide register–based cohort study
Source: Skin Health Dis. 2026 May 4;6(4):485–95. doi: 10.1093/skinhd/vzag054 (PMC13425034; doi:10.1093/skinhd/vzag054)
Supplement: vzag054_Supplementary_Data [file vzag054_supplementary_data.docx]

**Supplement Tables**

**Table S1 ATC codes and procedure codes of each drug used in psoriasis treatment**

| **Type of treatment** | **Drug substance** | **ATC code** | **Danish procedure code** |
| --- | --- | --- | --- |
| IL-12/23 | Ustekinumab | L04AC05 | BOHJ18B3 |
| TNFi | Etanercept | L04AB01 | BOHJ18A2 |
|  | Infliximab | L04AB02 | BOHJ18A1 |
|  | Adalimumab | L04AB04 | BOHJ18A3 |
|  | Certolizumab | L04AB05 | BOHJ18A5 |
| IL-17i | Sekukinumab | L04AC10 | BOHJ18B5 |
|  | Brodalumab | L04AC12 | BOHJ18B6 |
|  | Ixekizumab | L04AC13 | - |
|  | Bimekizumab | L04AC21 | - |
| IL-23i | Guselkumab | L04AC16 | BOHJ18B7 |
|  | Tildrakizumab | L04AC17 | - |

**Table S2 Drug survival by treatment in Bio-naïve population, 2018-2023**

| **Treatment** | **Treatment group** | **Drug survival rate (%) by time (years)** | | | | |
| --- | --- | --- | --- | --- | --- | --- |
|  |  | **1** | **2** | **3** | **4** | **5** |
| Ustekinumab | Ustekinumab | 43 | 30 | 23 | 17 | 15 |
| Adalimumab | TNFi | 25 | 9 | 4 | 2 | 1 |
| Certolizumab |  | 29 | 13 | 8 | 3 | - |
| Etanercept |  | 27 | 13 | 7 | 3 | 1 |
| Infliximab |  | 47 | 32 | 27 | 22 | 16 |
| Bimekizumab* | IL-17i | 33 | - | - | - | - |
| Brodalumab |  | 17 | 8 | - | - | - |
| Ixekizumab |  | 21 | 5 | 2 | - | - |
| Secukinumab |  | 29 | 11 | 4 | 2 | 1 |
| Guselkumab | IL-23i | 26 | 11 | 9 | - | - |
| Tildrakizumab |  | 9 | - | - | - | - |

* Bimekizumab was first used in Denmark in 2022 and had less than one year of follow-up. The maximum follow-up period of bimekizumab was 315 days in bio-naïve population.

**Table S3 Drug survival by treatment and treatment series, 2018-2023**

| **Treatment** | **Treatment group** | **Treatment series^a^** | **Drug survival rate (%) by time (years)** | | | | |
| --- | --- | --- | --- | --- | --- | --- | --- |
|  |  |  | **1** | **2** | **3** | **4** | **5** |
| Ustekinumab | Ustekinumab | Total | 42 | 27 | 19 | 14 | 11 |
|  |  | First | 44 | 29 | 21 | 14 | 11 |
|  |  | Second | 43 | 27 | 19 | 15 | 12 |
|  |  | Third | 42 | 26 | 20 | 15 | 11 |
|  |  | Fourth+ | 37 | 24 | 18 | 13 | 11 |
| Adalimumab | TNFi | Total | 23 | 9 | 4 | 2 | 1 |
|  |  | First | 23 | 9 | 4 | 2 | 1 |
|  |  | Second | 20 | 8 | 4 | 1 | - |
|  |  | Third | 22 | 9 | 4 | 2 | 1 |
|  |  | Fourth+ | 21 | 8 | 3 | 1 | 1 |
| Certolizumab |  | Total | 24 | 9 | 4 | 3 | 1 |
|  |  | First | 27 | 10 | 4 | 3 | - |
|  |  | Second | 20 | 7 | 5 | 4 | 2 |
|  |  | Third | 31 | 14 | 3 | - | - |
|  |  | Fourth+ | 17 | 6 | 4 | 2 | - |
| Etanercept |  | Total | 23 | 10 | 5 | 3 | 1 |
|  |  | First | 24 | 10 | 5 | 3 | 1 |
|  |  | Second | 25 | 11 | 6 | 3 | 2 |
|  |  | Third | 20 | 9 | 4 | 3 | 2 |
|  |  | Fourth+ | 22 | 10 | 5 | 2 | 1 |
| Infliximab |  | Total | 49 | 36 | 31 | 26 | 21 |
|  |  | First | 51 | 37 | 30 | 25 | 20 |
|  |  | Second | 48 | 33 | 27 | 27 | 22 |
|  |  | Third | 50 | 38 | 38 | 32 | 28 |
|  |  | Fourth+ | 46 | 36 | 31 | 30 | 21 |
| Bimekizumab* | IL-17i | Total | 19 | - | - | - | - |
|  |  | First | 41 | - | - | - | - |
|  |  | Second | 33 | - | - | - | - |
|  |  | Third | 30 | - | - | - | - |
|  |  | Fourth+ | 11 | - | - | - | - |
| Brodalumab |  | Total | 15 | 7 | 3 | 3 | - |
|  |  | First | 16 | 8 | - | - | - |
|  |  | Second | 17 | 8 | - | - | - |
|  |  | Third | 9 | 5 | - | - | - |
|  |  | Fourth+ | 15 | 7 | 6 | 6 | - |
| Ixekizumab |  | Total | 21 | 6 | 3 | 1 | 1 |
|  |  | First | 19 | 4 | 1 | 1 | - |
|  |  | Second | 19 | 4 | 1 | - | - |
|  |  | Third | 23 | 7 | 5 | - | - |
|  |  | Fourth+ | 22 | 8 | 4 | 2 | 2 |
| Secukinumab |  | Total | 29 | 11 | 5 | 2 | 1 |
|  |  | First | 26 | 8 | 3 | 1 | 1 |
|  |  | Second | 26 | 10 | 4 | 3 | 2 |
|  |  | Third | 35 | 18 | 10 | 5 | 3 |
|  |  | Fourth+ | 32 | 12 | 5 | 2 | 1 |
| Guselkumab | IL-23i | Total | 28 | 14 | 10 | 8 | 6 |
|  |  | First | 48 | 10 | - | - | - |
|  |  | Second | 41 | 28 | 19 | 19 | - |
|  |  | Third | 22 | 6 | 6 | - | - |
|  |  | Fourth+ | 26 | 14 | 10 | 8 | 6 |
| Tildrakizumab |  | Total | 6 | - | - | - | - |
|  |  | First | 0 | - | - | - | - |
|  |  | Second | 8 | - | - | - | - |
|  |  | Third | 0 | - | - | - | - |
|  |  | Fourth+ | 17 | - | - | - | - |

a. “Total” in Treatment series is same results in Table 2.

* Bimekizumab was first used in Denmark in 2022 and had less than one year of follow-up. The maximum follow-up period of bimekizumab was 349 days.

**Table S4 Log-rank tests for difference in drug survival depending on PsA status.**

| **Treatments** | **Treatment group** | **Subgroup^a^** | **Total numbers (N)** | **Observed** | **Expected** | **Deviation** | **χ^2b^** | **P-value** |
| --- | --- | --- | --- | --- | --- | --- | --- | --- |
| Ustekinumab | Ustekinumab | *With PsA* | 926 | 677 | 597 | 10.85 | 19.4 | 0.00001 |
|  |  | *Without PsA* | 1304 | 889 | 969 | 6.68 |  |  |
| Adalimumab | TNFi | *With PsA* | 9496 | 7825 | 8251 | 22 | 72.7 | < 0.00001 |
|  |  | *Without PsA* | 6336 | 5123 | 4697 | 38.7 |  |  |
| Certolizumab |  | *With PsA* | 739 | 630 | 636 | 0.0527 | 0.36 | 0.5 |
|  |  | *Without PsA* | 159 | 135 | 129 | 0.2593 |  |  |
| Etanercept |  | *With PsA* | 3715 | 3184 | 3206 | 0.145 | 1.02 | 0.3 |
|  |  | *Without PsA* | 755 | 660 | 638 | 0.728 |  |  |
| Infliximab |  | *With PsA* | 866 | 546 | 587 | 2.85 | 11.1 | 0.0009 |
|  |  | *Without PsA* | 360 | 251 | 210 | 7.96 |  |  |
| Bimekizumab | IL-17i | *With PsA* | 52 | 14 | 10.7 | 0.99 | 1.55 | 0.2 |
|  |  | *Without PsA* | 94 | 24 | 27.3 | 0.39 |  |  |
| Brodalumab |  | *With PsA* | 227 | 197 | 182 | 1.23 | 2.55 | 0.1 |
|  |  | *Without PsA* | 300 | 240 | 255 | 0.88 |  |  |
| Ixekizumab |  | *With PsA* | 1283 | 1009 | 1068 | 3.25 | 18.7 | 0.00002 |
|  |  | *Without PsA* | 468 | 367 | 308 | 11.26 |  |  |
| Secukinumab |  | *With PsA* | 2052 | 1747 | 1480 | 4.7 | 17 | 0.00004 |
|  |  | *Without PsA* | 1064 | 944 | 851 | 10.2 |  |  |
| Guselkumab | IL-23i | *With PsA* | 303 | 235 | 220 | 1.09 | 2.84 | 0.09 |
|  |  | *Without PsA* | 255 | 181 | 196 | 1.22 |  |  |
| Tildrakizumab |  | *With PsA* | 20 | 18 | 20.9 | 0.402 | 1.22 | 0.3 |
|  |  | *Without PsA* | 32 | 32 | 29.1 | 0.288 |  |  |

a. Without PsA: psoriasis-diagnosed patients not diagnosed with psoriatic arthritis (PsA). With PsA: psoriasis-diagnosed patients diagnosed with PsA.

b. One degree of freedom for all tests.

**Table S5 Detailed baseline characteristics**

| **Treatments** | **Treatment group** | **Subgroup^a^** | **Number of unique patients** | **Mean Age (Standard deviations)** |
| --- | --- | --- | --- | --- |
| Ustekinumab | Ustekinumab | *Total* | 1308 | 46.55 (16.74) |
|  |  | *With PsA* | 540 | 50.65 (15.77) |
|  |  | *Without PsA* | 793 | 44.07 (16.82) |
| Adalimumab | iTNFi | *Total* | 6694 | 48.98 (15.07) |
|  |  | *With PsA* | 4192 | 50.75 (14.34) |
|  |  | *Without PsA* | 2573 | 45.7 (15.83) |
| Certolizumab |  | *Total* | 455 | 48.02 (15.13) |
|  |  | *With PsA* | 370 | 48.36 (14.92) |
|  |  | *Without PsA* | 87 | 46.36 (16.03) |
| Etanercept |  | *Total* | 1857 | 53.46 (14.33) |
|  |  | *With PsA* | 1572 | 53.61 (13.83) |
|  |  | *Without PsA* | 295 | 52.62 (16.89) |
| Infliximab |  | *Total* | 1083 | 48.84 (14.58) |
|  |  | *With PsA* | 774 | 50.51 (13.42) |
|  |  | *Without PsA* | 315 | 43.44 (16.7) |
| Bimekizumab | IL-17i | *Total* | 140 | 51.17 (14.43) |
|  |  | *With PsA* | 51 | 53.04 (14.61) |
|  |  | *Without PsA* | 90 | 50.25 (14.3) |
| Brodalumab |  | *Total* | 262 | 47.99 (14.76) |
|  |  | *With PsA* | 113 | 50.33 (14.01) |
|  |  | *Without PsA* | 161 | 46.37 (15.06) |
| Ixekizumab |  | *Total* | 954 | 51.2 (13.87) |
|  |  | *With PsA* | 718 | 52.29 (13.71) |
|  |  | *Without PsA* | 245 | 46.57 (13.62) |
| Secukinumab |  | *Total* | 1312 | 49.12 (13.92) |
|  |  | *With PsA* | 929 | 50.83 (13.18) |
|  |  | *Without PsA* | 407 | 45.12 (14.75) |
| Guselkumab | IL-23i | *Total* | 269 | 49.91 (14) |
|  |  | *With PsA* | 140 | 51.56 (13.44) |
|  |  | *Without PsA* | 134 | 48.05 (14.38) |
| Tildrakizumab |  | *Total* | 18 | 42.58 (12.46) |
|  |  | *With PsA* | 8 | 38.41 (13.86) |
|  |  | *Without PsA* | 10 | 45.73 (10.36) |

a. Total: Total population regardless Psoriatic arthritis (PsA) status. Without PsA: psoriasis-diagnosed patients not diagnosed with PsA. With PsA: psoriasis-diagnosed patients diagnosed with PsA.

**Supplement Figure legends**

**Figure S1.** **Kaplan-Meier curves of the 6-year drug survival rates for each drug in Bio-naïve population.** Each drug is represented by a distinct color.

**Figure S2.** **Kaplan-Meier curves of the 6-year drug survival rates for each drug by treatment series.** Each drug is represented by a distinct color.
